# Estrogens and Androgens in Plants: The Last 20 Years of Studies

**DOI:** 10.3390/plants10122783

**Published:** 2021-12-16

**Authors:** Anna Janeczko

**Affiliations:** Polish Academy of Sciences, The Franciszek Górski Institute of Plant Physiology, Niezapominajek 21, 30-239 Kraków, Poland; ania@belanna.strefa.pl

**Keywords:** testosterone, androsterone, androstenedione, estradiol, estrone, plants

## Abstract

Although the only known steroid hormones in plants are brassinosteroids, interestingly, mammalian steroid hormones such as androgens or estrogens are also part of the plant metabolic profile. This presented review is focused on the progress that has been made in this matter during the last two decades. The presence of testosterone, 17β-estradiol, and other androgens/estrogens in plants (particularly those that can be measured using more advanced techniques) is described. The physiological activity of androgens and estrogens, especially in plants’ stress response, are discussed, together with some possible mechanisms of their action. The current knowledge indicates that although androgens and estrogens do not have the status of hormones in plants, they are physiologically active and can serve as regulators that support the activity of classic hormones in (1) regulating the various processes connected with plant growth and development and (2) the interaction of plants with their environment.

## 1. Introduction—Steroid Hormones in Living Organisms

Steroids are a group of compounds to which a number of crucial metabolism-controlling hormones belong. The group of steroid hormones that are present and active in animals and humans (mammalian steroid hormones) is large and includes, among others, corticosteroids, which control, for example, water and mineral management and sex hormones—i.e., androgens, estrogens, progesterone, which are responsible for development and reproduction [1,2,3]. On the other hand, ecdysteroids are mainly known as being the steroid hormones of arthropods that regulate ecdysis and development [4]. In plants, the steroid regulators include the brassinosteroids, which are hormones that have a multidirectional activity and are engaged in plant growth, development, and its response to environmental stresses [5,6]. Interestingly, however, mammalian steroid hormones are also part of the metabolic profile in plants [7,8]. While the debate about whether these compounds are also hormones for plants is still open, currently, there is a great deal of literature data to show that mammalian steroids influence the physiological processes of plants. There are review articles devoted to this issue that are available, starting from older ones such as Heftmann [9], Geuns [10], Hewitt et al. [11] to newer ones such as Janeczko and Skoczowski [12], Speranza [13], or Islam [14]. Since some of the recent reviews have been devoted to progesterone [15,16], this article is focused on the presence of/physiological activity of androgens and estrogens in plants and the progress that has been made in this field during the last 20 years.

Estrogens and androgens in animals and humans are sex steroid hormones with a multidirectional activity that includes the widely understood generative development and reproduction [17,18,19,20,21,22]. The best-known functions of androgens are the formation of the male sexual organs, the development of male sexual characteristics (body structure, voice, hair type, etc.), and their anabolic effects (increase in muscle mass, etc.). Estrogens regulate, among others, the formation of the female genital organs and breasts in prenatal life and after birth, shaping the psyche and sex drive, the menstrual cycle, lipid metabolism (HDL–LDL cholesterol balance, excretion of cholesterol in the bile, an increase the level of α-lipoproteins and phospholipids), calcium metabolism (i.e., the deposition of calcium in bones) and blood coagulability. Simultaneously, however, because of their proliferative activity, estrogens are connected with the stimulation of some cancers such as breast cancer [23]. Interestingly, in plants (for example, from the *Fabaceae* family) phytoestrogens (genistein, daidzein) are present. Due to their similar structure to estrogens, they can interact with human estrogen receptors and have many physiological activities in humans ([24] and literature cited therein).

Androgens are sometimes described as ‘male sexual hormones’, whereas estrogens are described as ‘female sexual hormones’, although they are produced (and active) by both sexes albeit in different amounts. The level of androgens and estrogens in a body depends on many factors, but on average, the daily production of testosterone in males is 6480 µg per day, while in females, it is 240 µg per day [25]. The daily production of estrogens (β-estradiol+estrone) in males is 140, while in females, it is about 630 µg per day.

**Figure 1:**
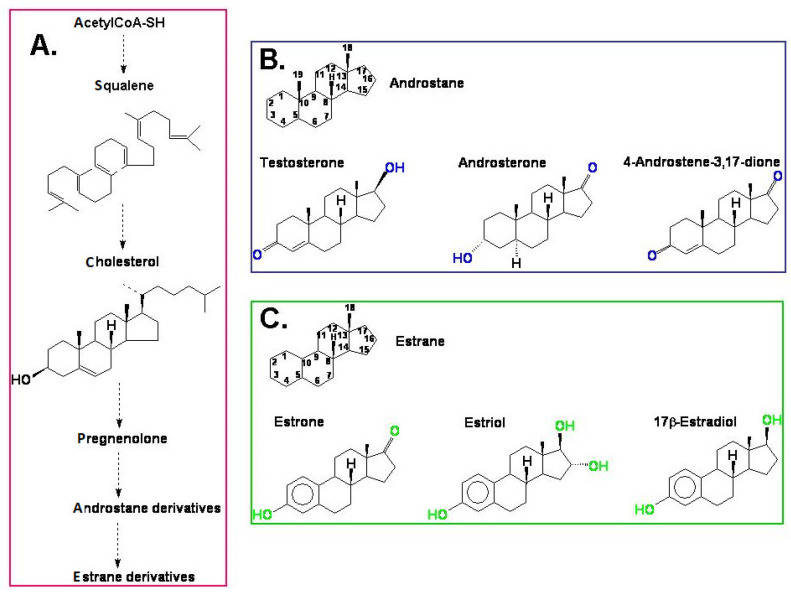
(**A**) A simplified model of the biosynthesis of steroid hormones (androstane and estrane derivatives) in animals and humans; (**B**) the chemical structure of androstane and selected androgens; (**C**) the chemical structure of estrane and the most important estrogens.

The androgens and estrogens in mammals are synthesised from cholesterol [26] via pregnenolone and progesterone and are built based on an androstane or estrane skeleton, respectively (Figure 1). Among androgens, the most well-known compound is testosterone. This group also includes 5α-dihydrotestosterone (DHT), androsterone, dehydroepiandrosterone (DHEA), and 4-androstene-3,17-dione (androstenedione). Androsterone and testosterone in animals and humans are the main precursors of another group of hormones—estrogens—and enzyme aromatase also participates in this biosynthesis, which is also called estrogen synthetase/synthase. Estrogens include 17β-estradiol, estrone, and estriol. The chemical structure of selected androgens and estrogens is presented in Figure 1. The biosynthetic pathway of steroid hormones (androstane and estrane derivatives), presented in Figure 1, is well characterised in animals and humans. We can only suspect that a similar pathway also functions in plants.

## 2. The Presence of Estrogens and Androgens in Plants: The Effect of Plant Growth Conditions and Plant Developmental Stage on the Steroid Content

According to the older literature data, estrogens were first found in plants as early as the 1930s [27], and then, for several decades, further studies documenting the presence of estrogens (and androgens) in plants were undertaken (reviewed in [12]). The development of analytical techniques in the last two decades has improved the detection of androgens and estrogens in plant material. This applies to improvements in methods of extraction and purification of compounds isolated from plant material (for example, the use of immunoaffinity columns with antibodies [28]), as well as detection methods (UHPLC–MS/MS and GC–MS/MS) [28,29,30]. There is also the possibility of using commercial kits for determining these compounds [31,32]. The presence of endogenous estrogens and androgens might be questioned, one reason of which is that these compounds are present in very small amounts, and at the same time, there are many other metabolic components in the plant extract that can hinder analysis and lead to false results. For this reason, in more advanced analytical methods, the step of appropriate cleaning of the sample should be emphasised. In the work of Simerský et al. [28], the sensitivity of the analysis is enhanced by including an immunoaffinity chromatography purification (using generic anti-Δ^4^-3-ketosteroid antibodies). These antibodies were used in the preparation of immunoaffinity gels for the purification and preconcentration of steroids in extracts. The use of immunoaffinity columns improved the sensitivity of UHPLC-ESI(+)-MS/MS measurement, and the so-called sample matrix effect was reduced and signal strength improved. In multiple-reaction-monitoring (MRM) mode, the detection limit for steroids was close to 10 fmol, and the response was linear up to 50 pmol injected. The MRM transitions from [M + H]^+^ ion to appropriate product ion provided precise quantification of the analysed steroids. The second MRM transition was also measured for each analyte to enable steroid conformation to be determined. The calculated MRM ratio was useful as another criterion based on which an analyte may be distinguished from interfering substances. More details about the procedure can be found in [28].

During the last 20 years, several papers that documented the occurrence of androgens and estrogens have been published. The compounds in question are present in plants at the pg and ng levels. The impact of plant growth conditions and the plant developmental stage on the steroid content was also confirmed. In some cases, it has even been possible to correlate the changes in the concentration of estrogens and androgens with specific physiological processes.

Khaleel et al. [33] studied the 17β-estradiol distribution during the development and expression of the reproductive structures in *Populus tremuloides*. The amount of the hormone varied from 14 pg/g F.W. × 10^−1^ in a bisexual tree (branch 1, November) to 2624 pg/g F.W. × 10^−1^ in the reproductive buds of a bisexual tree (March). Generally, the authors found that in catkins, the hormone concentrations were higher before anthesis, peaked during flowering, and then lowered when the flowers matured. According to the authors, the increases were accompanied by sporogenesis and the development of gametophytes. Moreover, a seasonal variation in the 17β-estradiol concentration was observed, as well as the impact of light conditions and the plant organ being studied. Dormant winter tissues contained a lower 17β-estradiol content than spring tissues. Branches that were growing in more intense light had a higher 17β-estradiol content. Changes in 17β-estradiol and testosterone were also observed in kiwifruit pollen by Speranza et al. [34]. Both hormone levels increased during pollen germination—namely, during the phases of tube organisation, emergence, and subsequent elongation. The content of 17β-estradiol in kiwifruit pollen varied from almost 0 (in ungerminated pollen) to about 4 ng/mg pollen after 90 min of germination, while the testosterone content varied from 0 to 2.5 ng/mg pollen. The application of bisphenol A additionally increased the levels of 17β-estradiol and testosterone. As concentrations of exogenous 17β-estradiol and testosterone in medium that were too high inhibited kiwifruit pollen germination, environmental bisphenol A contamination might also be a cause of the disruption in the fertility of a plant via its impact on the steroid content. According to other authors [29,32], the content of estrogens and androgens in plants in some cases might also be dangerous for humans and animals. Lu et al. [29] tested eight species—lettuce, tomato, pumpkin, potato, carrot, citrus, apple, and strawberry—and found that the concentration of 17β-estradiol in these vegetables and fruits ranged from 1.3 to 2.2 ng/g F.W. The exceptions were tomato and strawberry, in which no 17β-estradiol was detected. A second estrogen, estrone, was present in the tested material in an amount of less than 0.8 ng/g (except for lettuce, tomato, and strawberry, in which it was not detected). The authors noted that the estimated daily intake of 17β-estradiol for children might be above the recommended acceptable daily intake. Presence of estrogens and androgens in food (including foods of plant origin) was also reviewed by Palacios et al. [35].

The presence of estrogens and androgens in plants, in some circumstances, might also be a problem for animals. Zeitoun and Alsoqeer [32] studied the sex steroid hormones in alfalfa and in some rangeland native species in Saudi Arabia and their subsequent effects on camel reproduction. Testosterone was found in *Cakile arabica* (3.69 ng/g D.W.) and *Cyperus conglomerates* (2.97 ng/g D.W.) but not in *Plantago boissieri*, *Rhanterium epapposum*, *Halexylon salicornicum*, *Heliotropium bacciferum*, *Cenchrus ciliaris*, and *Medicago sativa.* Additionally, 17β-estradiol was found in *Lactuca serriola* (379 pg/g D.W.), *Eruca sativa* (247 pg/g D.W.) and *H. bacciferum* (229 pg/g D.W.) but not in *Leptadenia pyrotechnica*, *R. epapposum*, and *Stipagrostis pulmosa*. According to the authors, because of the presence of these hormones, among others, camels could suffer from cystic ovarian syndrome and delayed pregnancy.

Milanesi et al. [36] detected estrogens (17β-estradiol and estrone) in the seeds, leaves, flowers, and calli of *Solanum glaucophyllum*. The steroid concentration was dependent on the tissue/organ. In fact, 17β-estradiol was found in all of the aforementioned tissues, with its highest level in seeds—120 ng/kg F.W. Estrone was found in the calli and seeds (only a few ng/kg F.W.) but not in the flowers or leaves. Milanesi and Boland [37] also reported the presence of estrogen-like compounds in the leaves of tomato plants.

As was mentioned above, Khaleel et al. [33] found that there are seasonal variations in the steroid level in plants. One of the causes might be the impact of growth temperature on the steroid level. Janeczko et al. [38] found that androgen (4-androstene-3,17-dione [androstenedione]) was present in the leaves of wheat that was growing at 20 °C (about 20 pg/g F.W.), and that its content decreased fourfold after the plants were exposed to cold (5 °C).

Androstenedione was also found in the leaves of *Nicotiana tabacum* and *Inula helenium* (about 8 and 11 pmol/g F.W., respectively) but not found in *Digitalis purpurea* [28]. According to Tarkowská [8], testosterone and androst-4-ene-3,17-dione were detected in *Tribulus terrestris* in a range of 0.01–0.05 ng/g F.W.

As was mentioned at the beginning of this section, all of the results show that the estrogens and androgens that were confirmed in plants in amounts of pg and ng usually referred to g of fresh weight (Table 1); definitely, the highest concentrations of androgen and estrogen was found in pollen. In some species, however, the content of these steroids has not yet been confirmed, which does not mean that they are not present in much lower amounts (below the detection limits). In the future, further development of analytical methods might enable the trace amounts of these steroids to also be measured in plant material. This would be necessary, especially in a case in which these steroids would gain the status of plant hormones in the future. Additionally, there is also a necessity to confirm pathways of biosynthesis of androgens and estrogens in plants and to find molecular biology evidence to support them. This is especially needed to rule out the possibility that estrogens or androgens in plants may be the result of the action of inhabiting microorganisms. Androgen-producing microorganisms are known as being a part of the human microbiome [39]. On the other hand, it is also possible that part of estrogens/androgens can be produced in plant cells, and part by plant-inhabiting microorganisms. This phenomenon is known in plants for classic hormones—for example, cytokinins [40] or gibberellins.

Nevertheless, combining the changes in the steroid content with specific physiological or morphological changes [33,34] led us to the next issue—the physiological activity of estrogens and androgens in plants.

## 3. Physiological Activity of Estrogens and Androgens in Plants

### 3.1. Plant Growth and Reproduction

#### 3.1.1. Plant Growth

The impact of estrogens and androgens on plant growth was already described in the first half of the 20th century (reviewed by [12]). In the last 20 years, few new works have been published that describe the effects of these hormones on plant growth, development, or other processes, as well as revealing some of the mechanisms of their action in detail. Interestingly, however, in recent years, there has been a number of works that have been devoted to the study of the impact of these mammalian hormones on plants due to the fact that they are found in waste or sewage and thus are ‘artificially’ introduced into the environment [41,42,43,44]. In these works, the authors show that high concentrations of estrogens have a harmful effect on plant growth, morphology, and development. According to Adeel et al. [44], in lettuce, the application of estrogen (17β-estradiol 10 mg/L) significantly reduced the total root growth and development, which was connected to an increased accumulation of H_2_O_2_, higher lipid peroxidation, and a higher activity of the antioxidant enzymes. Moreover, 17β-estradiol (at the same concentration), when applied to corn, inhibited kernel germination and corn seedling growth [43]. The studies of Brown [42] were devoted to determining the effects of mammalian estrogens (17β-estradiol, estrone, and estriol) on the growth and tuberisation of potato plants (*Solanum tuberosum* L.). At a concentration of as low as 0.1 mg/L, estrogen reduced root growth, while 10 mg/L of estrogen caused plant deformities and induced a callus. Tuber production was slightly lower in the plants to which estrogen had been applied, compared with the control. Estrogens at a concentration of 10 mg/L also lowered the activity of an enzyme (acid phosphatase) that is important in plant mineral management, etc.

Although these reports proved that at high concentrations, estrogens have an inhibitory effect on the growth processes in plants, simultaneously, at lower concentrations, they might have a biostimulative effect on metabolism. In lettuce, estrogens that were applied at concentrations of 0.1–50 µg/L enhanced the photosynthetic pigments, root growth, and shoot biomass [44]. In addition, 17β-estradiol (10^−6^ M) stimulated the accumulation of the photosynthetic pigments in *Wolffia arrhizal* [41]. Dumlupinar et al. [45] found that a 10^−6^ M concentration of 17β-estradiol and androsterone, when applied to seven-day-old barley seedlings via spraying, increased the concentrations of calcium, magnesium, phosphorus, sulphur, copper, manganese, aluminium, zinc, iron, potassium and chlorine most effectively, while they decreased the sodium concentration in barley leaves (measured 18 days after spraying). Further, 17β-estradiol (at a concentration of 0.1 mg/L) stimulated germination and seedling growth in corn [43]. According to Brown [42], however, even at 0.1 mg/L, estrogens reduced root growth in potato plants although the acid phosphatase activity of the plants increased. In chickpea (*Cicer arietinum*) plants, a concentration of 17β-estradiol and androsterone 10^−9^ M (applied to seven-day-old plants) was the most active in stimulating plant growth, which was connected with an increased protein and sugar content 18 days after spraying [46]. The contents of mineral elements such as K, S, Na, Ca, Mg, Zn, Fe, P, Cu, and Ni were higher, whereas Mn and Cl were lower [47]. The same concentration of these steroids also effectively lowered the H_2_O_2_ content and lipid peroxidation along with a higher activity of the antioxidative enzymes [46]. Erdal and Dumlupinar [48] studied also the effects of 17β-estradiol on germination, root and shoot growth, and the biochemical background (among the other activity of *α*-amylase) in chickpea. The seeds, which had been germinated at a few concentrations of steroid from 10^−4^ to 10^−15^ M were then analysed at the end of the 1st, 3rd, and 5th days. Based on the results, 17β-estradiol accelerated seed germination at the end of days 1 and 3, and the root, and shoot growth was also stimulated. The most effective concentrations of 17β-estradiol were in the range of 10^−9^–10^−12^ M. These effects were accompanied by an increase in the activity of *α*-amylase during germination. The effect of 17β-estradiol, estrone, and androsterone on the in vitro regeneration of *Triticale* mature embryos was described by Uysal and Bezirganoglu [49]. Estrogens had the best result on the percentage of explants that formed shoots.

#### 3.1.2. Plant Reproduction

The impact of estrogens and androgens on plant development (some aspects such as sex expression) was described before 2000 (reviewed in [12]). The regulatory effect of estrogens and androgens on sex expression in plants was, however, also critically reviewed by Jones and Roddick [50]. In the last 20 years of studies, this aspect of steroid activity was rather abandoned and other effects of steroids on generative development were emphasised.

In some ornamental plants (*Petunia hybrida*, *Tagetes erecta*, and *Calendula officinalis*), 17β-estradiol (1 mg/L) increased the leaf area but not the flowering longevity [51]. Żabicki et al. [52], on the other hand, studied the effect of estrone (1 and 3.7 µM) on the differentiation of the somatic tissues and on the induction of an autonomous endosperm in a culture of female gametophyte cells of *Arabidopsis thaliana*. Estrone stimulated the development of an autonomous endosperm in unpollinated pistils, direct organogenesis, callus proliferation, and the formation of trichome-like structures (‘hairs’). Histological analysis revealed adventitious root formation. Rojek et al. [53] examined the effect of exogenous estrone and androsterone on an unfertilised egg and central cell divisions in a culture of unpollinated pistils of *A. thaliana* wild-type and *fie* mutants. Both steroids stimulated the central cell divisions and fertilisation-independent endosperm development. The stages of autonomous endosperm development were similar to the pattern that was observed after fertilisation. Importantly, the developmental arrest of the autonomous endosperm at the nuclear stage was overcome by the application of the steroid, as the switch from the nuclear to the cellular stage of the endosperm is required for the correct embryo and seed development. In the *fie* mutants, which inherited the autonomous underdeveloped endosperm (more about *fie* in [54]), the steroids clearly accelerated and promoted the development of the full *fie* autonomous endosperm. The changes in the methylation of the *FIE* gene (*FERTILIZATION-INDEPENDENT ENDOSPERM* gene; [54]) were established in in vitro conditions [53], which suggests that full autonomous endosperm development could be a synergistic effect of changes in the histone modification and DNA methylation within a distinct set of common target genes that are involved in endosperm development [53,55,56].

The effect of estrogens (estrone, estriol, 17β-estradiol) and androgens (androsterone, androstenedione) on the generative induction of *A. thaliana* and winter wheat was described by Janeczko and Filek [57] and Janeczko et al. [58]. *A. thaliana* is a plant with a photoperiodical flowering control (it needs long-day conditions to induce a bloom). Winter wheat (especially the cultivar Grana), on the other hand, has a thermoperiodical control of development, which means that it needs a sufficiently long cold period (usually a few weeks) to induce the generative stage. In the case of both species, failure to meet these conditions (or their fulfilment in an incomplete manner, for example, too short of a cold period for wheat) results in generative developmental disorders, delayed flowering, or even suppressed flowering. Hence, both plants are useful models to study the activity of the regulatory compounds in relation to the stimulation of generative development because it can be determined whether they replace the action of the physical factors such as the length of the day (*A. thaliana*) or the cold period (winter wheat). Janeczko et al. [58] found that *A. thaliana* responded (in relation to the induction of generative development) to the application of estrogens and androgens differently in vitro. The absolute control plants (grown under long-day conditions throughout the growing season) reached a 100% generative phase. Only 41 percent of the plants that were only allowed 7 days of growth under long-day conditions (second control) reached the generative phase. Androsterone and androstenedione (at a concentration of 0.1 μM), when applied in the same growth conditions as the second control, had the opposite effect on the generative development of *A. thaliana* than the estrogens did. The androgens increased the percentage of generative plants up to 90–96%, respectively (almost to the level noted in the absolute control). The estrogens at the same concentration decreased the number of generative plants to as little as 0 (in the case of estrone). On the other hand, a higher concentration (1 µM) of estrone increased the percentage of plants in the generative stage by up to 80%, 17β-estradiol by almost up to 70%, while estriol yielded results that were similar to the second control. Androgens at a concentration of 1 µM also stimulated generative development; however, in the case of androsterone, it was less effective than at a concentration of 0.1 µM. In winter wheat, higher concentrations (1 and 10 µM) were used because the plants only grew in the in vitro culture for a short, initial life period (compared with *A. thaliana*, in which hormone exposure lasted throughout the entire period of vegetation) [57]. After 28 days of vernalisation (cold treatment), the heading stage reached 100% of the plants that had been treated with androgens at both concentrations (plants untreated with steroids—0%). In the case of estrogens, similar to in *A. thaliana*, the higher concentration was more effective.

To summarise, experiments using exogenous estrogens and androgens have shown a wide spectrum of their effects on plant growth and development. Unfortunately, it is currently not possible to confirm the physiological activity of estrogens and androgens in plants in tests on mutants with disturbances in the biosynthesis of these compounds (such mutants are not available to the best of the author’s knowledge). However, an alternative (and chance) is to use the inhibitors of androgen/estrogen biosynthesis implemented from medical sciences, as was carried out in the case of progesterone [59].

### 3.2. Plant Stress Response

Compared with the studies on the activity of estrogens and androgens on the growth and development of plants, which, as was mentioned above, had already begun in the first half of the 20th century, research on the anti-stress activity of these compounds has mainly been performed in the last 20 years. One of the reasons for this is the fact that during this period, the general research interest in the mechanisms of the plant stress response increased. During their growth in the environment, plants are exposed to many stress factors—abiotic (a deficit or excess of water, a too low or high temperature, salinity, heavy metals) and biotic (pathogens). Their impact on plant yield has become more and more important from an economic point of view in light of the increasing world population. Many researchers are searching for/studying the antistress-protecting regulators that might alleviate plant stress. For steroids, these studies are mainly focused on the plant hormones, brassinosteroids (extensive literature is available), although mammalian steroid hormones have also been of research interest. The effects of estrogens/androgens on the metabolism of crop plants (mainly from the cereal group and from the *Fabaceae* family), under low-temperature stress, drought, salinity stress, and heavy metal stress have been studied [15,38,60,61,62,63].

#### 3.2.1. Low-Temperature Stress

Maize is a cold-sensitive species, in which even short periods of cold (10/7 °C (day/night) for three days) cause stress injuries [60]. The foliar application of androsterone (10^−9^ mol/L) reduced the detrimental effects of low temperature in maize seedlings [60]. In cold-stressed seedlings, the oxidative damage (measured as malondialdehyde level) was less in the androsterone-treated seedlings, which was accompanied by an increase in the activity of the antioxidant enzymes such as superoxide dismutase or catalase and ascorbate peroxidase. Moreover, the isozymes of superoxide dismutase and ascorbate peroxidase (gel electrophoresis) exhibited a correlation with changes in their activity. Androsterone also increased the content of ascorbic acid, glutathione, proline, and carotenoids. Androsterone reduced the negative effects of cold on the chlorophyll content and also reduced electrolyte leakage (which provides information about cell membrane permeability). The effect of androsterone on membrane permeability is particularly interesting in light of the findings of Janeczko et al. [38] for winter wheat. Unlike maize, winter wheat is a species for which cold is naturally required for acquiring tolerance to more extreme temperatures during winter— namely, frost. Cold-treated plants adjust their hormonal, sugar, and lipid management in order to be more frost tolerant. A higher level of sugars prevents cell rupture due to the freezing of water and an increase in the percentage of unsaturated fatty acids is beneficial for the flexibility and fluidity of cell membranes in cold. In the studies of Janeczko et al. [38], the aim was to investigate the physicochemical and biochemical background of the activity of an androgen (androstenedione) in winter wheat that had been exposed to a low temperature. Wheat seedlings (control and androgen-supplemented) were cold hardened (14 days) and then exposed to freezing temperature (−12 °C). Steroid supplementation reduced the number of frost injuries to the leaves by 30%. A Langmuir bath analysis showed that androstenedione was absorbed into the plant cell membranes. It increased the distance between the lipid molecules in membranes, which increased fluidity and might be important for an androgen-induced tolerance to frost. Additionally, androstenedione accelerated the generative development of the wheat stimulating accumulation among other gibberellins and cytokinins. In the aerial part of the plants, the level of the gibberellic acid GA_3_ was increased by 30% and cytokinin *cis*-zeatin by 65%, compared with the control. The content of other hormones (auxins, abscisic acid and salicylic acid) was also changed in a manner dependent on plant organ or time of cold treatment [38]. Based on these results, we cannot exclude the fact that estrogens and androgens (at least in part) act in plants indirectly via regulation of biosynthesis of classic plant hormones.

Interestingly, despite the positive effects of exogenous androstenedione on the frost tolerance of wheat, its endogenous level decreased during cold hardening [38]. That is why it is difficult to say whether this compound really matters in acquiring frost tolerance in these species.

#### 3.2.2. Drought and Salinity Stress

Janeczko et al. [64] studied the possibility of compensating for the negative effects of drought stress on the gaseous exchange and efficiency of PSII in soybean by applying androstenedione (0.25 mg/L, presowing seed soaking). The steroid improved the intensity of leaf net photosynthesis, but the effect was not directly visible during drought, only visible during the rehydration of the plants that had undergone a period of drought. An increase in the net photosynthesis intensity was accompanied by higher transpiration. The authors discussed the hypothesis that possible mechanisms of androstenedione action might be connected with its effect on aquaporin functionality and membrane stability.

In wheat seedlings, both 17β-estradiol and androsterone (applied at concentrations 10^−6^ to 10^−10^ mol/L via leaf spraying) increased salt tolerance [61]. The salt-stressed and steroid-treated plants accumulated more dry weight and were capable of synthesising more sugars and proteins than the plants that had not been treated with 17β-estradiol and androsterone. The steroids promoted the accumulation of proline and glutathione. The activities of the antioxidant enzymes and nitrate reductase were also higher. Both steroids compensated for the salt-induced chlorophyll losses. In wheat, the steroids were the most effective at a concentration of 10^−8^ mol/L. In maize, on the other hand, 17β-estradiol and androsterone reduce the salt-stress effects most effectively at concentrations of 10^−8^ and 10^−10^ mol/L, respectively [62]. In maize seedlings, these two steroids mitigated the salt-induced growth inhibition (both in the roots and aerial parts). Similar effects on the metabolism were observed as in the case of wheat.

#### 3.2.3. Heavy Metal Stress

The protective effect of 17β-estradiol (application via seed soaking) against the toxicity of heavy metals (cadmium and copper) in *Lens culinaris* was studied by Chaoui and El Ferjani [63]. Estrogen limited the heavy metal-induced decrease in dry weight accumulation but did not reduce the accumulation of cadmium and copper in the tissues. Rather, it modulated the cellular biochemistry in the direction of mobilisation of the antioxidant system and limited the peroxidation of the membrane lipids similar to what was observed earlier in the case of salt or drought stress.

#### 3.2.4. Biotic Stress

The effects of 17β-estradiol on growth, primary metabolism, phenylpropanoid-flavonoid pathways, and pathogen resistance in *Arabidopsis thaliana* were studied by Upadhyay and Maier [65]. Estrogen-treated plants were inoculated with *Pseudomonas syringae* pv. tomato DC3000 and had a higher susceptibility to this pathogen than the control. Low concentrations of estrogen (10 and 100 nM), however, stimulated root growth and shoot biomass accumulation and increased the efficiency of photosynthesis and the accumulation of sugars and proteins. A higher concentration (10 µM) inhibited plant growth. Notably, 17β-estradiol generally caused the downregulation of the phenylpropanoid-flavonoid pathway genes (*PAL1*, *PAL4*) and decreased the level of phenolics, flavonoids, and anthocyanins.

**Figure 2:**
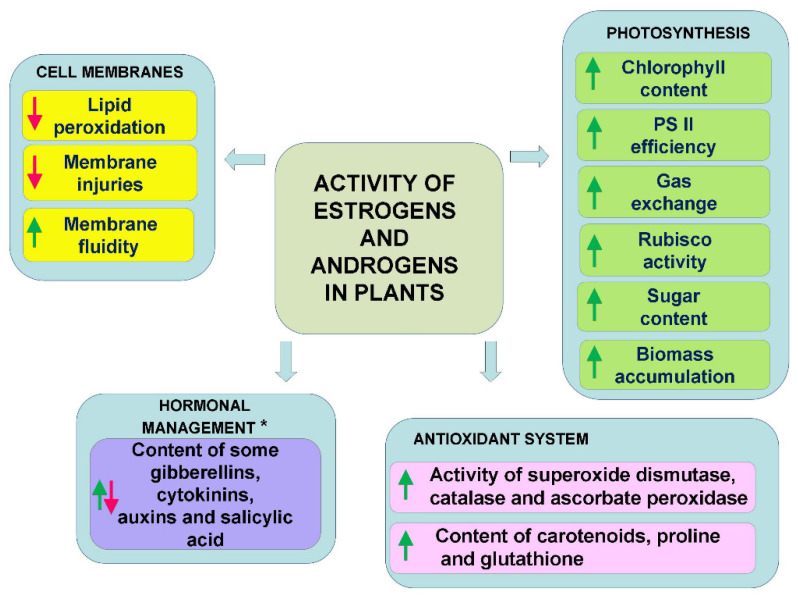
General directions of activity of estrogens and androgens (applied at low concentrations) in plants exposed to abiotic stresses. *—For detailed information, see Section 3.2.1. Plant Stress Response, which noted the dependency on plant organ (aerial part, roots) and time of exposition to stress factors.

In conclusion, as can be seen from the literature discussed above, all of the works indicate that estrogens and androgens regulate metabolism, thereby influencing photosynthesis and sugar production, the antioxidant system, the properties of the cell membranes, and the synthesis of various plant pigments (chlorophyll, carotenoids, anthocyanins) (Figure 2). In the case of abiotic stresses, this is also accompanied by an improvement in tolerance to these stresses. Interestingly, however, the action of the typical environmental stresses is not the only factor that induces the mobilisation of the antioxidant system or changes in the membranes by estrogens or androgens. Many authors have also observed such an effect in plants that had not been exposed to stress [49,51,66,67]. This could indicate that these steroids induce some metabolic reactions in plants that could prepare (harden) plants for potential future stress. On the other hand, it can also be interpreted differently—i.e., that the administration of these hormones itself is a mild stress for plants, which mobilises their metabolism.

As mentioned in the Section 1, in plants steroid hormones are present—namely, brassinosteroids. There are considerable similarities in the activity of brassinosteroids and androgens/estrogens in plants exposed to stresses (improvement of efficiency of photosynthesis, effect on sugar production, stimulation of the antioxidant system, regulation of the properties of the cell membranes, and stimulation of photosynthetic pigment production)—compare scheme in a review study of brassinosteroids by Sadura and Janeczko [5]. The question that should be asked is whether this is due to the somewhat similar structure of brassinosteroids and estrogens/androgens and is related to binding to the same steroid-binding proteins (receptors). Perhaps estrogens/androgens also ‘use’ signalling pathways of brassinosteroids. As for the effect of steroids on membrane fluidity, it is also known that compounds with a structure similar to sterols penetrate membranes and modulate their properties (hence the effects observed in Langmuir bath experiments for both brassinosteroids and estrogens/androgens [38,68]).

Finally, in animals and humans are generally two main mechanisms of action of steroid hormones—genomic (slow, involving gene expression) and nongenomic (for example, fast cellular calcium exchange) [69,70,71]. We can hypothesise that similar mechanisms are also present in plants, but this will require further studies. An example of the genomic effect of estrogens could be the downregulation of the phenylpropanoid–flavonoid pathway genes (*PAL1*, *PAL4*) by 17β-estradiol [65]. Nongenomic effects (in the case of androstenedione) could be somehow connected with alterations in membrane fluidity/permeability induced by androstenedione [38].

In the future, the use of plants with impaired biosynthesis of estrogens/androgens (mutants or transgenic plants) or experiments using inhibitors of biosynthesis or action of estrogens/androgens should be performed. Such experiments would be useful to elaborate/confirm the mechanism of action and functions of the steroids mentioned above.

## 4. Transport and Conversion of Estrogens and Androgens in Plants

Important elements of the physiological activity of various regulators are their uptake and accumulation in plant tissues. The research that has been conducted to date, which has shown that estrogens and androgens can be absorbed by plants, was conducted mainly because the environment may be contaminated with these compounds (animal-based waste, manure). Nevertheless, they provided information that plants are able to uptake steroids, and their accumulation (greater or lesser) influences the physiological processes in a dose-dependent manner [44]. The uptake of 17α-ethynylestradiol in bean plants (*Phaseolus vulgaris*) was confirmed by Karnjanapiboonwong et al. [72]. An accumulation of 17α-ethynylestradiol was detected in the roots and leaves. In plants that were grown in sand (conditions of high contaminant bioavailability), the accumulation was higher than in plants that were grown in soil. The shoots and roots of maize that had been exposed to hydroponic solutions containing 2 μM 17β-estradiol and estrone also accumulated these compounds [73]. Estrogens were found in the roots at concentrations of up to 0.19 μmol/g; the highest level was after 1–3 days of exposure. Only 17β-estradiol was accumulated in the shoots. The authors also suspected a transformation and/or irreversible binding processes of estrogens in plant tissues. In another study, [74] proved oxidative (17β-estradiol to estrone) and reductive (estrone to 17β-estradiol) transformations. The combined effects of plant enzymes and plant-associated microbes were responsible for those transformations. The uptake and possibilities of converting estrogens were also studied in a culture of lettuce [44]. Two estrogens—17β-estradiol and ethinyl estradiol—were administrated to plants at concentrations of 0, 0.1, 50, 150, 2000, and 10,000 µg/L via the roots (nutrient medium). The uptake was dose dependent, and a higher level of the applied estrogens was detected in the leaves and roots. In control plants, estrogens were not found. Biotransformation of applied estrogens was noted. In the case of the roots of lettuce, after the application of 17β-estradiol (lower concentration), estrone was also detected. The application of higher concentrations of 17β-estradiol also resulted in an accumulation estrone and 17α-estradiol. In the case of the leaves, both compounds were detected no matter which concentration was applied. After the application of ethinyl estradiol in the roots and leaves (especially when a higher concentration was used) ethinyl estradiol as well as estrone, 17β-estradiol, and 17α-estradiol were detected.

As for the androgens, data are available for 17β-trenbolone. Trenbolone belongs to synthetic androgens that have anabolic properties. Blackwell et al. [75] described the uptake and biotransformation of 17β-trenbolone in *Phaseolus vulgaris*. In this study, 17β-trenbolone was biodegraded to trendione (less active androgen) in vegetated sands (microbial degradation). In plants, the trenbolone metabolites were primarily concentrated in the roots, and only small concentrations were moved to stem and leaves.

To conclude, plants are able to uptake/transform estrogens and androgens. The microbial enzymatic systems also participate in the biotransformations of estrogens and androgens [74,75].

## 5. Receptors (Specific Binding Sites) of Estrogens and Androgens in Plants

Estrogen and androgen receptors in animals and humans are well known and described, although new discoveries are still being made in this regard. It can generally be assumed that there are three groups of these receptors—membrane, nuclear, and cytoplasmic [76,77,78,79,80,81].

To date, the knowledge about the putative estrogen receptors in plants is very limited, although it seems that a similar division of estrogen-binding proteins (membrane, nuclear, and cytoplasmic) also exists in the plant kingdom. In plants, Milanesi et al. [36] and Milanesi and Boland [37] described the presence of putative estrogen receptors (estrogen-binding sites) in *Solanum glaucophyllum* and *Lycopersicon esculentum*. Experiments were performed using [^3^H]17β-estradiol. The *S. glaucophyllum* callus also contained (except for the natural estrogens) estrogen-binding sites [36]. More in-depth studies showed that the estradiol-binding capacity can be found in the soluble fractions of the roots, stems, and leaves of *S. glaucophyllum* and *L. esculentum.* The range was from 100 to more than 3000 fmol/mg of protein and was dependent on the organ (the highest was in roots, and the lowest was in leaves). Western and ligand blot analyses suggested that the estrogen-binding proteins in plants may be present in a nuclear fraction, mitochondrial fraction, microsomes, and cytosol [37]. The presence of specific binding sites in the cytosol and membrane fraction was also described by Janeczko et al. [82] for winter wheat (up to more than 40 fmol/mg protein). The membrane and cytosol fractions of non-vernalised (control) and vernalized (cold-treated) plants were tested using a tritium-labelled ligand. The specific binding of [^3^H]17β-estradiol was detected, and it was generally higher in the membranes than in the cytosolic fraction. The specific binding of ligand was dependent on the plant growth conditions and was higher in the membrane fraction in the control than in the cold-exposed plants. Due to the positive effect of estrogens on stimulating the development of winter wheat [57], it is likely that these specific binding sites of 17β-estradiol are engaged in the mechanism of wheat development, but this will require further studies.

To the best of the author’s knowledge, there are no reports about androgen-specific binding sites in plants, and therefore, this matter still requires further study to find an explanation.

## 6. Concluding Remarks

Although estrogens and androgens have been studied in terms of their physiological activity and their presence in plants for much longer than brassinosteroids, it is the brassinosteroids that have been recognised as plant hormones. In the last 20 years, the progress in brassinosteroid research has been tremendous, compared with studies of the activity/role of estrogens and androgens in plants. There are known various brassinosteroid mutants, brassinosteroid receptor is well characterised, biosynthetic pathways and signal transduction is described, and the common presence of brassinosteroids in the plant kingdom is proved. To the best of the author’s knowledge, the pathway of biosynthesis of estrogens and androgens in plants is still unclear. There are no mutants with disturbances in the biosynthesis of estrogens and androgens in plants, and the inhibitors of their biosynthesis that are known from medical or veterinary sciences have not been used in experiments on plants. This additionally makes it difficult to elaborate/confirm the role of estrogens and androgens in plants. Nevertheless, analytical studies show that estrogens and androgens are present in plants. The current state of knowledge allows us to suggest that estrogens and androgens can be considered to be a kind of ‘supporting regulators’ for plant metabolism (relative to the classic plant hormones such auxins or gibberellins). However, in the future, further research on estrogens and androgens in plants will surely reveal new facts about their importance and their role in the plant kingdom.

## Figures and Tables

**Table 1:** Exemplary results of analysis of estrogens and androgens in plants (last 20 years). For better comparison of results, original units were recalculated/unified to pg per g of plant material (if necessary). F.W.—fresh weight; D.W.—dry weight.

Species	Steroids	Original Units	Units [if Recalculated to pg per g]	Reference
*Populus tremuloides*	17β-estradiol	14 pg/g F.W. × 10^−1^ in a bisexual tree (branch 1, November) 2624 pg/g F.W. × 10^−1^ in the reproductive buds of a bisexual tree (March)	-	[33]
Kiwifruit	17β-estradiol	up to 4 ng/mg pollen (dependent of stage of germination)	up to 4,000,000 pg/g pollen	[34]
Kiwifruit	testosterone	0–2.5 ng/mg pollen (dependent of stage of germination)	0 to 2,500,000 pg/g pollen	[34]
Lettuce, pumpkin, potato, carrot, citrus, apple	17β-estradiol	1.3–2.2 ng/g F.W.	1300–2200 pg/g F.W.	[29]
Pumpkin, potato, carrot, citrus, apple	estrone	less than 0.8 ng/g F.W.	less than 800 pg/g F.W.	[29]
*Cakile arabica* *Cyperus conglomerates*	testosterone	3.69 ng/g D.W. 2.97 ng/g D.W.	3690 pg/g D.W. 2970 pg/g D.W.	[32]
*Lactuca serriola* *Eruca sativa* *Heliotropium bacciferum*	17β-estradiol	379 pg/g D.W. 247 pg/g D.W. 229 pg/g D.W.	-	[32]
*Solanum glaucophyllum*	17β-estradiol	120 ng/kg F.W. (seeds) 4–10 ng/kg F.W. (calli, leaves, flowers)	120 pg/g F.W. (seeds) 4–10 pg/g F.W. (calli, leaves, flowers)	[36]
*Solanum glaucophyllum*	estrone	3–6 ng/kg F.W. (calli and seeds)	3–6 pg/g F.W. (calli and seeds)	[36]
Winter wheat	androstenedione	21.7 pmol/g F.W. (leaves of seedlings growing at 20 °C)	6215 pg/g F.W. (leaves of seedlings growing at 20 °C)	[38]
*Nicotiana tabacum* *Inula helenium*	androstenedione	7.69 pmol/g F.W. (leaves) 11 pmol/g F.W. (leaves)	2177 pg/g F.W. 3202 pg/g F.W.	[28]

## Data Availability

Data is contained within the article.
